# Position Weight Matrix or Acyclic Probabilistic Finite Automaton: Which model to use? A decision rule inferred for the prediction of transcription factor binding sites

**DOI:** 10.1590/1678-4685-GMB-2023-0048

**Published:** 2024-01-19

**Authors:** Guilherme Miura Lavezzo, Marcelo de Souza Lauretto, Luiz Paulo Moura Andrioli, Ariane Machado-Lima

**Affiliations:** 1 Universidade de São Paulo, Instituto de Matemática e Estatística, Programa Interunidades de Pós-Graduação em Bioinformática, São Paulo, SP, Brazil.; 2 Universidade de São Paulo, Escola de Artes, Ciências e Humanidades, São Paulo, SP, Brazil.

**Keywords:** Transcription factor binding site, position weight matrix, ChIP-seq, position dependency, model comparison

## Abstract

Prediction of transcription factor binding sites (TFBS) is an example of application of Bioinformatics where DNA molecules are represented as sequences of A, C, G and T symbols. The most used model in this problem is Position Weight Matrix (PWM). Notwithstanding the advantage of being simple, PWMs cannot capture dependency between nucleotide positions, which may affect prediction performance. Acyclic Probabilistic Finite Automata (APFA) is an alternative model able to accommodate position dependencies. However, APFA is a more complex model, which means more parameters have to be learned. In this paper, we propose an innovative method to identify when position dependencies influence preference for PWMs or APFAs. This implied using position dependency features extracted from 1106 sets of TFBS to infer a decision tree able to predict which is the best model - PWM or APFA - for a given set of TFBSs. According to our results, as few as three pinpointed features are able to choose the best model, providing a balance of performance (average precision) and model simplicity.

## Introduction

Embryo development, cancer and stem cell differentiation are examples of biological processes regulated by transcription regulation (Xiao *et al.*, 2018; Furlong and Levine, 2018; Andersson and Sandelin, 2019). These complex mechanisms require understanding how *cis*-regulatory modules (CRM) affect expression of gene regulatory cascades. CRMs are DNA sequences upstream or downstream of the target gene where multiple transcription factors (TFs) can bind and trigger mechanisms that can increase or decrease gene expression (Spitz and Furlong, 2012). Furthermore, TFs recognize and bind to specific short DNA sequences (usually 6 to 12 nucleotides) called transcription factor binding sites (TFBS) and different TFs recognize different patterns of DNA sequences to bind (called motifs) (Lambert *et al.*, 2018). Therefore, it is important to know which TFs bind to a given CRM and the location of their binding sites to associate a given target gene with its regulators. In this article, we limit our scope to TFBS recognition in high throughput sequencing experiments. More specifically, how to choose an appropriate model that can predict the pattern of recognition of a given TF.

State-of-the-art molecular biology techniques to uncover TFBSs include chromatin immunoprecipitation sequencing (ChIP-seq), chromatin immunoprecipitation on chip (ChIP-chip) and protein binding microarray (PBM). Table 1 shows additional information about these techniques.

**Table 1 -:** Biological techniques to uncover TFBSs.

Techniques	Description
ChIP-seq	ChIP-seq, a powerful in vivo technique investigates TF-DNA interactions along the whole genome (Landt *et al.*, 2012; Nakato and Sakata, 2021). The technique sequences several DNA fragments of approximately 150-500 nt-long that were bound to a specific TF. These sequences are aligned to the source genome to identify the location of the statistical relevant “peaks” of these mapped sequences (Nakato and Sakata, 2021). These peaks, with hundreds to few thousands of nucleotides in length, are the most probable regions containing the binding sites of that TF.
ChIP-chip	ChIP-chip is a technique similar to ChIP-seq where instead of the immunoprecipitated DNA being sequenced, it is hybridized into a microarray chip 6. Each spot emits a fluorescence signal when DNA hybridization occurs to quantify the signal intensity and identify “peaks” containing the TFBSs (Lee *et al.*, 2006). The length of these peaks ranges from hundreds to few thousands of nucleotides.
PBM	Protein binding microarray (PBM) is an *in vitro* technique that identifies which of several artificially generated random DNA sequences are the most recognized by a TF of interest (Berger and Bulyk, 2009). PBM provides higher resolution results but is less reliable than in vivo techniques, because this assay does not take into account all biological events that simultaneously happen during TF binding to DNA.

However, these biological experiments uncover sequences containing the TFBSs but not their exact location. Frequently, an additional step is necessary for motif discovery, in order to identify short similar subsequences shared by these sequences (Kulakovskiy and Makeev, 2009; Boeva, 2016).

In addition to the resolution issue, these biological experiments are expensive and time consuming. Therefore, performing these experiments in all genomes and for all TFs are often very expensive. Computational prediction of TFBSs is an important strategy to identify high resolution binding site locations in a faster and cheaper manner.

The most used model for TFBS prediction is the *Position Weighted Matrix* (PWM) (Staden, 1984). A PWM is a matrix *W* where *W*_*ij*_ contains the score associated with the occurrence of the nucleotide *i* = 1,..,4 (representing *A*, *C*, *G* and *T*) in the position *j* of the binding site of a specific TF. The score of a sequence *x = x*_1_…*x*_*l*_ is the sum of the scores *W*_*ij*_ for the nucleotides present at each position *j =* 1,…,*l*. If the score of *x* is above a predefined threshold, *x* is considered a binding site for that TF. It is a simple and easy-to-train model that achieves good results (Wasserman and Sandelin, 2004; Zhao and Stormo, 2011). However, PWMs are based on the assumption that the occurrence of a nucleotide in a certain DNA’s position does not depend on the presence of these nucleotides in its vicinity, which is not necessarily true. In fact, some studies suggest the existence of positional dependencies between nucleotides within TFBSs (Tomovic and Oakeley, 2007; Badis *et al.*, 2009; Zhao *et al.*, 2012; Eggeling *et al.*, 2014). One possible explanation is the idea that the TFs recognize not only nucleotide composition, but also the structure of the DNA sequence (Badis *et al.*, 2009; Slattery *et al.*, 2014; Schnepf *et al.*, 2020). Notwithstanding the evidence for such dependencies, it is still an open question whether this is true for all TFs or even for a particular family of TFs (Badis *et al.*, 2009; Zhao *et al.*, 2012; Weirauch *et al.*, 2013).

**Figure 1 -:**
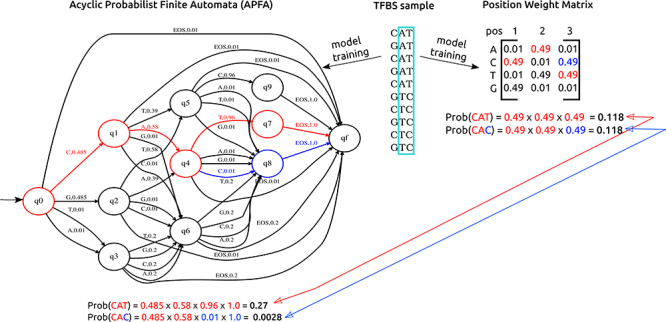
Example of models trained from a pseudo TFBS sample in order to show how APFA (left) and PWM (right) models estimate their probability parameters and how dependencies are, or not, considered. For the sake of simplicity and with no impact in the comparison, the illustrated APFA and PWM scores were calculated based on equation 1 with pseudocount = 0.1, ie, before the division by the null model probabilities and log calculation. In the APFA, circles represent analysis states that are followed left-to-right during the analysis of an input sequence. Each edge is labeled with a nucleotide or EOS (“end of sequence”), and a probability. The sum of the probabilities of all edges going out of each state is one. Here, the PWM matrix has at position (i, j) the probability of a nucleotide i is present at TFBS position j. The green box in the TFBS sample shows a dependency between nucleotide positions 2 and 3. Whereas APFA was able to represent such dependency, PWM was not able. For instance, the symbol “T” in the third position of the sequence “CAT” has a high probability (0.96) in the APFA once it appeared after “CA” (path shown in red), whereas “C” in the third position of the sequence “CAC” has a low probability (0.01) also due to their previous nucleotides (last part of the path shown in blue). However, PWM ascribes the same probability of 0.49 to each nucleotide “T” or “C” in the third position.

Assuming that the TFBS motifs have a fixed length, such inter-position dependencies can be modeled by an *acyclic probabilistic finite automata* (APFA) (Ron *et al.*, 1998). Figure 1 depicts the ability of this model to represent conditional position dependence and the inability of PWMs for this purpose. The question is: *as APFA is a more complex model than PWM, will it always outperform PWMs or only under certain conditions?*

In this article, we answered this question by not only comparing the results using PWMs and APFAs for the problem of binding site prediction for several TFs, but also inferred a decision rule to choose which model to use based on features extracted from the training sequences, such as conditional position dependence measures. In addition, we analyzed the influence of different motif discovery methods to identify the training sequences from biological experiments. It is a relevant issue because a motif discovery algorithm may consider, or not, possible existence of conditional position dependence.

### Position Weight Matrix

Position Weight Matrices are matrices *W*_*4×l*_ that characterize motifs of length *l*. Each position *W*_*ij*_ contains a score associated with the occurrence of the nucleotide *b*_*i*_ , *i* = 1,..,4 (*b*_1_ = *A*, *b*_2_ = *C*, *b*_3_ = *G* and *b*_4_ = *T*) in position *j* of the TFBS motif (*b*_*ij*_ ). Such score is based on the log of the likelihood ratio of a trained TFBS model p^ and a null model q. A typical null model in Genomics corresponds to the nucleotide frequencies in the genome of study.

More precisely, we define p^bij as:

p^bij=nij+ψbin+∑i=14ψbi, (1)

where n_*ij*_ is the absolute frequency of the nucleotide b_*i*_ at position j in the TFBS training sample, $\psi \left(b_{i}\right)$ is the value of a pseudocount function for b_*i*_ , which helps smoothing probabilities, avoiding zero value in p^bij (Wasserman and Sandelin, 2004), and n is the number of sequences in the TFBS training sample.

The pseudocount $\psi \left(b_{i}\right)$ is arbitrary, but defined here as:

$$\psi \left(b_{i}\right)=\alpha f_{bi}$$ (2)

with $\alpha =\frac{1}{10n}$ , a proportionally inverse of the TFBS sample size (n) and f_*bi*_ the absolute frequency of b_*i*_ in the TFBS sample (Xia, 2012).

Finally, the score W_*i,j*_ of a PWM is defined as:

Wi,j=log2p^bijqbi, (3)

where q(b_*i*_ ) is the b_*i*_ probability according to the null model.

Let s = s_1_s_2_…s_*l*_ be a sequence with length l and s_*j*_ is the specific nucleotide occurring in that position. Therefore, we define i(j) = 1,2,3,4 if the s_*j*_ = A,C,T,G, respectively, i.e., s_*j*_ = b_*i(j)*_ . A PWM ascribes a score W(s) as:

$$W\left(s\right)=\sum_{j=1}^{l}W_{i\left(j\right),j}$$ (4)

### Acyclic Probabilistic Finite Automata

APFAs are a subclass of Probabilistic Finite Automata, that are devices from formal language theory which consists of a set of states and state transition rules of stochastic nature, defined on an alphabet of symbols, able to attribute probabilities to the recognized sequences. Informally, an APFA is an automaton where states are organized in levels from the start state q_0_ to the final state q_*f*_ , and all edges connects the state from one level to a state in the next level (as illustrated in the left side of Figure 1) or to the final state q_*f*_ (Ron et al., 1998). Therefore, APFAs with levels l + 1 are suitable for modeling the distribution of sequences with a maximum length of l. An algorithm for APFA learning (structure and probabilities) is described in (Ron et al., 1998).

The probability P_*A*_ (s) assigned to a sequence s = s_1_s_2_…s_*l*_ by an APFA A is the product of each state transition rule used to generate s. Similar to PWMs, we also calculated the log-odd score:

$$H_{A}\left(s\right)={log}_{2}\left(P_{A}\left(s\right)\right)-{log}_{2}\left(q\left(s\right)\right)$$ (5)

Where q(s) is the probability of s evaluated by a null model:

$$q\left(s\right)=\prod_{i=1}^{l}q\left(s_{i}\right)$$ (6)

### Measures of inter-position dependency

Two different methods used in this work to measure inter-position dependencies in TFBS samples are Cramér’s V (or Φ) and Theil’s U. The advantage of these methods in comparison to others used in this context (Tomovic and Oakeley, 2007) is that Cramér’s V and Theil’s U have a fixed range of values (from zero to one), allowing to compare different TFBS samples.

These methods calculate the dependency between two positions in a set of sequences. For this, they assume B is a random variable that can take values in {A,C,T,G}, and b_*j*_ is a particular value (nucleotide) of B in position j. P(b_*j*_ ) is the probability of B at position j taking value b_*j*_ , and P(b_*h*_ ,b_*j*_ ) is the joint probability of B taking value b_*j*_ at position j and b_*h*_ at position h.

Cramér’s V

Derived from χ^2^ (Chi-square) statistics, Cramer’s V is a measure of association between two categorical variables, with zero meaning no association and one meaning total correlation (Kim, 2017). We define χ^2^_*jh*_ , the χ^2^ statistics for positions j and h, as:

$$\chi_{jh}^{2}=n\sum_{b_{j},b_{h}}\frac{\left(P\left(b_{j},b_{h}\right)-P\left(b_{j}\right)P\left(b_{h}\right)\right)²}{P\left(b_{j}\right)P\left(b_{h}\right)}$$ (7)

Where n is the number of sequences in the sample.

Finally, we define V_*jh*_ as:

$$V_{jh}=\sqrt{\frac{\chi_{jh}^{2}}{min\left(c-1,r-1\right)n}}$$ (8)

where χ^2^_*jh*_ is defined in equation 7, and c and r are the number of columns and rows, respectively, in the contingency table (c = r = 4 for TFBS samples.)

Symmetric Theil’s U

Symmetric Theil’s U (named as Theil’s U here and after) is a normalization of Mutual Information (I), taking values ranging from zero to one (Witten et al., 2011). Let I(B_*j*_ , B_*h*_ ) be the Mutual Information of B_*j*_ and B_*h*_ , calculated as:

$$I\left(B_{j},B_{h}\right)=\sum_{b_{j}}\sum_{b_{h}}P\left(b_{j},b_{h}\right)log\left(\frac{P\left(b_{j},b_{h}\right)}{P\left(b_{j}\right)P\left(b_{h}\right)}\right)$$ (9)

Then, U(B_*j*_ , B_*j*_ ), the Theil’s U of random variables B_*j*_ and B_*h*_ , is defined as:

$$U\left(B_{j},B_{h}\right)=2\left(\frac{I\left(B_{j},B_{h}\right)}{H\left(B_{j}\right)+H\left(B_{h}\right)}\right)$$ (10)

Where I(B_*j*_ , B_*h*_ ) is the Mutual Information defined in equation 9 and H(B_*j*_ ) is the entropy of B_*j*_ , defined as:

$$H\left(B_{j}\right)=-\sum_{b_{j}\in \{A,C,T,G\}}P\left(B_{j}\right)logP\left(B_{j}\right)$$ (11)

### Information content

Information Content (IC) is commonly used to measure the quality of a PWM (Xia, 2012). It is defined as:

IC=∑i=14∑j∈{1,2,..,k}p^bij×Wi,j (12)

with W_*i,j*_ defined in equation 3, p^bij defined in equation 1, i = 1,2,3,4 is each PWM row (representing nucleotides A,C,G and T, respectively), and j is each PWM column (representing the TFBS positions).

## Material and Methods

**Figure 2 -:**
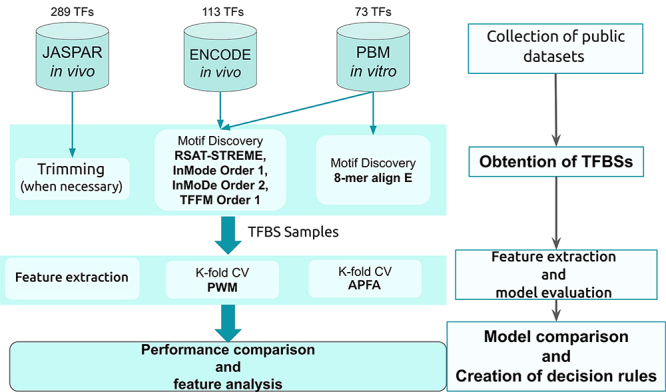
Summary of the employed workflow.

In order to create a decision rule to help choosing which model to use - PWM or APFA - based on a TFBS sample, we performed the strategy described in Figure 2. First, TFBS sequences were obtained using different motif discovery tools. Then, the TFBS samples were used for: 1) feature extraction and 2) estimation of the performance of PWMs and APFAs. Finally, these results were used to create the decision rule. In the following sections we give details of each process.

### Datasets

All datasets were collected from JASPAR, ENCODE and PBM databases. From JASPAR we downloaded, for each TF, the most recent version of ChIP-chip or ChIP-seq file that had 100 or more sequences. From ENCODE, files were downloaded via Bioconductor ENCODExplorer package, using the option “optimal IDR thresholded peaks,” for genomes mm10, hg19, dm6 and ce11. From PBM, both 8-mer (DNA sequences of length eight) processed data and raw data were downloaded from the project website (Weirauch et al., 2013).

In total, 289 TFBS samples were obtained from JASPAR, 113 from ENCODE and 73 from PBM experiments. Each TFBS sample is composed of TFBS sequences from a specific TF from a specific species.

### Data processing and motif discovery

JASPAR sequences are already processed by a motif discovery step. The exact TFBS sequences were obtained trimming lowercase characters. JASPAR uses uppercase to describe the exact TFBS sequence and lowercase to describe the upstream and downstream sequences. For ENCODE and PBM data, different motif discovery algorithms were applied, resulting in the creation of different TFBS samples.

There are a variety of available algorithms for motif discovery. Basically, they could be divided as considering or not conditional position dependencies. As this issue may impact the creation of the TFBS samples, we picked a spectrum of motif algorithms that could represent all ranges of assumptions about inter-position dependencies.

For motif discovery algorithms that consider dependencies we used TFFM (Mathelier and Wasserman, 2013) and InMoDe (Eggeling, 2018), which are based on variations of Markov Models. Algorithms that do not consider dependencies are RSAT (Nguyen et al., 2018) and STREME (Bailey, 2021), both of which are enumeration approaches that return a PWM as result. Additionally, the algorithm 8-mer align E can be seen as a control case regarding dependencies, since it is not based on a model, but solely on PBM signal-to-noise statistics over 8-mers (Berger and Bulyk, 2009; Weirauch et al., 2013).

We independently applied more than one motif discovery tool to each experimental dataset (e.g.: ENCODE ChIP-seq, JASPAR, Weirauch et al. (2013) available PBM data), resulting in different TFBS samples for each tool applied (see Figure 2). We did not merge TFBS samples obtained from the same experiment. Instead, we treated each sample as an independent dataset for the 7-fold cross-validation of both models (see section Material and Methods - Nested k-fold CV and Data S3).

In summary, 1106 TFBSs samples were obtained as a result of combining a dataset and a motif discovery tool: 289 TFBS samples from JASPAR, 113 × 4 = 452 TFBS samples from ENCODE and 73 × 5 = 365 TFBS samples from PBM (see Figure 2).

Details about the motif discovery protocol are described in Data S1 and Data S2.

### TFBS sample feature extraction

For each TFBS sample, dependence features were extracted based on Cramér's V and Theil’s U measures (see section Introduction - Measures of inter-position dependency). Since both measures are defined in terms of a pair of positions, we calculated the maximum and the mean value of each measure among all position pairs: max_Cramér’s V, mean_Cramér’s V, max_Theil’s U and mean_Theil’s U. In addition, for Cramér’s V and Theil’s U calculation (equations 8 and 10), we calculated P(b_*j*_ , b_*h*_ ) and P(b_*j*_ ) as follows.

Consider a TFBS sample of n sequences of length l. Let h and j be two arbitrary positions in a sequence, with h = 1,2,…, l and j = 1,2,…, l and h ≠ j. Let B be a random variable that can take values in {A, C, T, G}, and b_*j*_ a particular value (nucleotide) of B in position j.

The joint probability of b_*j*_ and b_*h*_ is defined as:

$$P\left(b_{h},b_{j}\right)=\frac{N\left(b_{h},b_{j}\right)+\frac{1}{n}}{n+\frac{16}{n}}$$ (13)

where N(b_*h*_ , b_*j*_ ) is the absolute frequency of the joint occurrences of b_*h*_ in position h and b_*j*_ in position j.

To avoid computation inconsistencies due to zero values in probabilities, we added a pseudocount of $\frac{1}{n}$ to each N(b_*j*_ , b_*h*_ ). As there are sixteen combinations of the pair N(b_*j*_ , b_*h*_ ), the denominator should be normalized as 16n as well.

Then, P(b_*j*_ ) is calculated as the marginal probability of B in position j taking the value b_*j*_:

$$P\left(b_{j}\right)=\sum_{b_{h}}P\left(b_{j},b_{h}\right).$$ (14)

Other features were also extracted: the number of sequences in the TFBS sample, and the mean Information Content (mIC) - a normalization of the IC (see section Introduction - Information Content) over the TFBS length l:

$$mIC=\frac{IC}{l}$$ (15)

### Model performance evaluation

We implemented a nested K-fold Cross Validation (CV) for the purpose of training and testing the models PWM and APFA, with K = 7, and applied to each of the 1106 positive TFBS samples (S^+^) obtained as described in section Material and Methods - Data Processing and Motif Discovery. As PWM and APFA models are trained using only positive sequences, non-positive sequences are used exclusively for testing. All tasks that involved some randomness were performed using Python pseudo-random numbers generator (random module) and seed set to 11.

Non-positive samples

We artificially generated sequences which were shuffled versions of the corresponding genome (of the species of the specific TFBS sample) in order to compose the “non-positive” sample, used only for testing. We use the term “non-positive” instead of “negative” sample because we cannot certify that shuffled sequences are not particular instances of motifs from a given TF target of study. The generation was performed following these steps:

1. using the corresponding genome of each TFBS sample, approximately 16,000 random DNA fragments were selected out of 1,000 nucleotides;
2. each fragment was shuffled (using Python random module) and sliced, where each “sliced” sequence had the same size of the TFBSs in S^+^;
3. because in the genome there are more “non-positive” sequences than actual TFBS for a specific TF, the final S¯ is composed of min(500,000; 100 × n), where n is S^*+*^ sample size.

Nested k-fold CV

**Figure 3 -:**
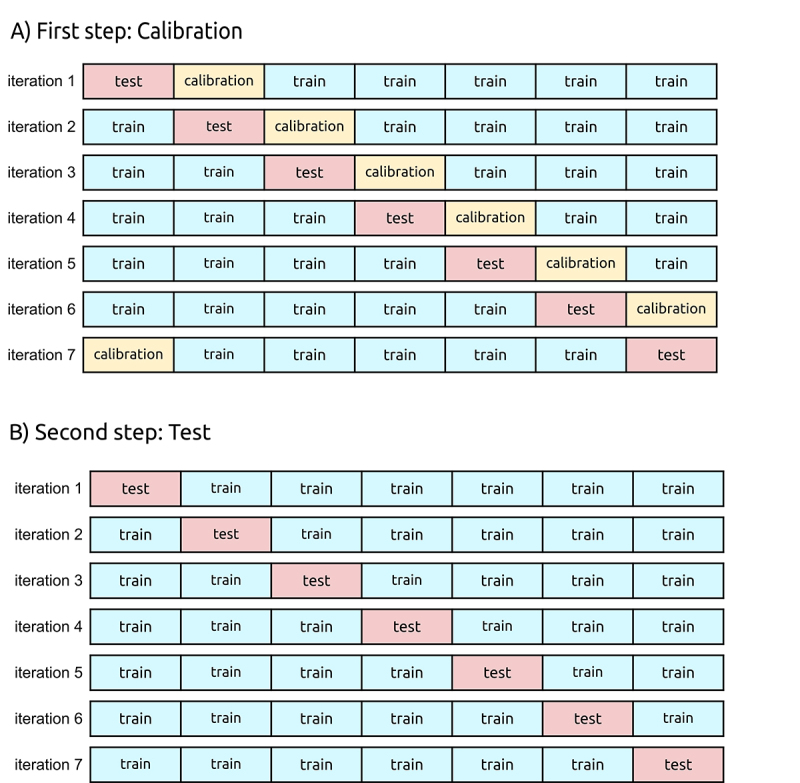
Nested 7-fold Cross-Validation. The whole TFBS sample is represented as a bar, and each fold is represented as a disjoint subset (rectangles). In A) the first step is illustrated where, in each iteration, train folds (in blue) are used to train the APFA and PWM models, and the calibration fold (in yellow) is used to calibrate the Learn-APFA hyperparameters and find the optimal classification thresholds for APFA and PWM. In this phase the test fold (in red) is not used. In B) the second step is illustrated, which estimates the performance of the APFA calibrated in the previous step and of the PWM, using the threshold values also calibrated in the previous step. In this phase, calibration fold is included in the training folds and the trained APFA and PWM models are applied to the test fold (positive sample in red, and additional negative samples). The final performance is the average of the performance values calculated in each iteration.

We used a 7-fold CV strategy divided in two nested steps illustrated in Figure 3 and summarized in Data S3 (Tables S1 and S2). The purpose of the first step (Figure 3A) or “inner loop” is to optimize 1) the hyperparameters of the Learn-APFA algorithm (Ron et al., 1998) used to train the APFAs (see details below) and 2) the classification thresholds for APFA and PWM models. 

We call hyperparameters the adjustable parameters of the learning algorithm used to guide the training process. The hyperparameters are not the learned values but can affect the overall performance of the model. APFA has three hyperparameters to be calibrated:

1. mu (μ): this parameter is directly involved in the generalization capacity of the model. It is an adjustable threshold used by the learning algorithm to consider if two subsequences are similar. When two subsequences are considered similar, the learning algorithm merges the corresponding internal states in the APFA, avoiding overfitting;
2. m_0_: this is another threshold that determines if the count of each subsequence in the training set is enough to be evaluated as similar according to the mu parameter mentioned above. Therefore, m_0_ verifies if there is enough statistical evidence in data to merge distinct states. This is important to control the generalization of the model, complementing the mu parameter;
3. gamma (γ): this parameter can be seen as the pseudocount value given to each nucleotide at a given position in case there is no observable count in the training set. This assures that no probability zero is given to a nucleotide during the classification task, which could lead to computational issues during the scoring of the whole sequence being evaluated.

APFA optimal hyperparameter combination is found using a grid search strategy, where the best combination is such that maximizes the Average Precision (AP) score calculated over the calibration folds, averaged over all iterations. Average Precision score is an estimator of Area Under the Precision Recall Curve (PRC). Precision Recall Curve and performance metrics were computed with scikit-learn (Pedregosa et al., 2011).

In addition, the optimal threshold that classifies a sequence in TFBS or non-TFBS can be also considered a hyperparameter. Therefore, the optimal thresholds for APFA and PWM models are also calibrated in this first cross-validation. For each model, the threshold is calculated as the average of the threshold values that maximize the F1-score calculated on the calibration folds.

In the second step (Figure 3B ) or “outer loop” the calibration fold integrates the training set to train the PWM model, as well as the APFA model using the optimal hyperparameters calculated in the first step. In addition, optimal threshold values for PWM and APFA, also calculated in the previous step, are used to estimate the PWM and APFA performance on test samples. Since PWMs and APFAs are learned from positive samples only, additional negative samples are also used to evaluate the models. The negative samples (Sˉ) were also split in 7 folds, each one used in an “outer loop” iteration of this nested 7-fold cross-validation. The model’s performance is estimated based on the AP score over the test samples (positive and negative). This average AP score is used for model comparison, as described in section Material and Methods - Model Performance and Evaluation, topic Model comparison and model preference prediction.

Model comparison and model preference prediction

For each specific TFBS sample, the model presenting the highest average AP, calculated (as described in section Material and Methods - Model performance evaluation, topic Nested k-fold CV), was considered the best model. The question is: is it possible to predict which model will be the best one for a specific TFBS sample based on some of its features, including particularly dependence features?

Sometimes both PWM and APFA present very similar performances.

Therefore, to answer the previous question, we transformed the problem of choosing a model by categorizing the PWM/APFA models for a given TFBS sample with Cohen’s D measure, which can discriminate between similar and non-similar AP values.

For each TFBS sample, let $\overset{¯}{{AP}_{PWM}}$ and $\overset{¯}{{AP}_{APFA}}$ be the average AP of PWM and APFA respectively. Cohen’s D, an effect size measure, is then defined as:

$$D=\frac{\;\overset{¯}{{AP}_{APFA}}-\;\overset{¯}{{AP}_{PWM}}}{s}$$ (16)

where s is the pooled standard deviation, defined as:

$$s=\sqrt{\frac{\left(n-1\right)s_{1}^{2}+\left(n-1\right)s_{2}^{2}}{2n-2}}$$ (17)

where s_*i*_^2^ is the variance of AP values over all folds (in k-fold Cross-Validation) for each model i, with i∈{PWM,APFA} and n is the number of AP values for each i, which corresponds to the number of folds in 7-fold Cross-Validation (n = 7).

Comparing the values, D ≥ 0.4 was found corresponding to the cases where AP^ˉ^_*APFA*_ was greater 5% or more than AP^ˉ^_*PWM*_ . Moreover, we found no significant improvement of PWM over APFA under any circumstances. Therefore, we defined this 0.4 threshold, where D ≥ 0.4 means APFA was the best performed model, and D < 0.4 means APFA and PWM were similar. Due to PWM simplicity when compared to APFA, we recommend PWM when D < 0.4.

Finally, the model preference was transformed into a binary classification problem, where each TFBS sample was considered an instance represented by its features (extracted as described in section Material and Methods - TFBS sample feature extraction) and class = 1 if its D ≥ 0.4 and 0 otherwise. This new data was visualized using Principal Component Analysis (PCA) and also used to train a Decision Tree to infer a prediction rule for the model preference.

To measure the performance of this decision tree, a stratified 10-fold CV was used, reporting the accuracy averaged over all ten iterations.

## Results and Discussion

### Impact of motif discovery tool on distribution of extracted features and model performance

We investigated and confirmed the hypothesis that distinct motif discovery tools applied in the same sequences can result in different TFBS samples with different dependence features. Therefore, we used the TFBS samples resulting from all these motif discovery tools in order to have a broader range of dependence feature values to compare in which conditions APFA is preferred over PWM or vice-versa.

Figure S1 presents the distributions of the extracted feature values for each category of TFBS (database and used motif discovery tool). As expected, for features based on dependency measures, we observed higher median values for samples originating from motif discovery algorithms that consider inter-position dependencies - InMoDe and TFFM - than those originated by algorithms that do not consider dependencies - JASPAR and RSAT-STREME. The mean_Cramér’s V and mean_Theil’s U values (Figure S1top) were considerably low, with median values ranging from 0.05 to 0.25 approximately. However, as shown in Figure S1 middle, the max_Cramér’s V and max_Theil’s U of each TFBS sample is considerably higher than their corresponding mean-based features. Together, these results suggest that, in general, there are few specific pairs of positions that are strongly dependent, but the dependency is low for most pair positions.

In addition, mIC values were higher when the used motif discovery tool does not consider position dependencies (Figure S1 bottom), which was also expected. Higher mIC values are expected when the sample sequences are more conserved (similar) in each particular position. Conversely, lower mIC values can indicate a more complex relationship between nucleotide positions, which independent models such as PWM cannot model properly.

Additionally, we analyzed the direct comparison between APFA and PWM AP values divided by each TFBS sample category (Figure S2). It can be observed that APFA performed better than PWM in motif discovery tools that use position-dependencies. This is evidenced by the fact that no point (i.e. TFBS sample) is below the diagonal line. In tools that do not use such dependencies, PWM and APFA perform similarly.

### PCA and decision rules

**Figure 4 -:**
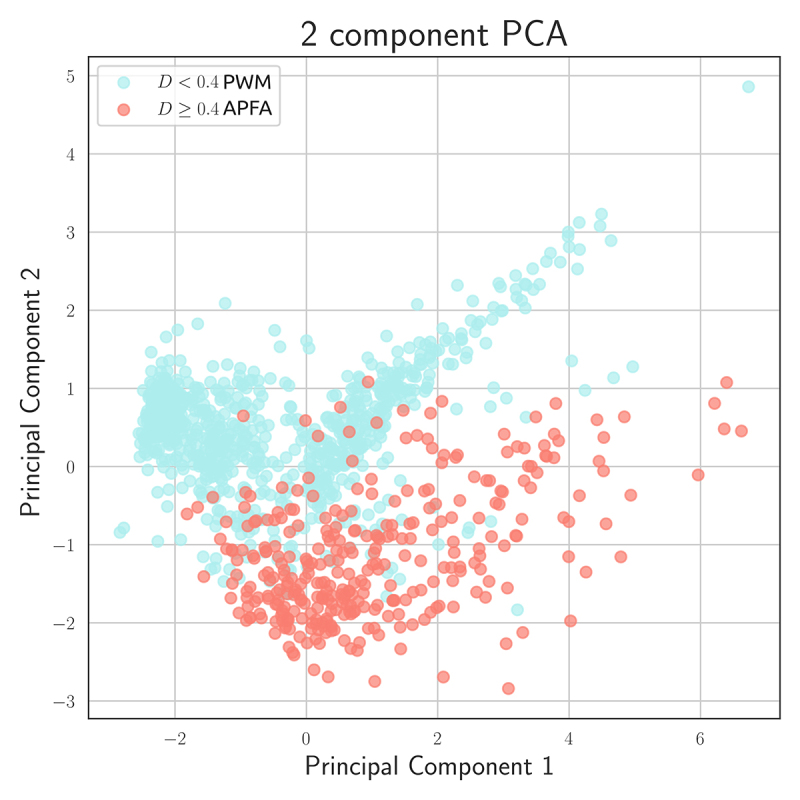
Principal Component Analysis using the extracted feature for model preference categories.

Figure 4 shows a PCA plot with two principal components using the five features considered in this work and the Cohen’s D categories previously used. Despite the mixture of categories near the boundaries, there is a solid distinction between categories D ≥ 0.4 (APFA preference) and D < 0.4 (PWM preference).

**Figure 5 -:**
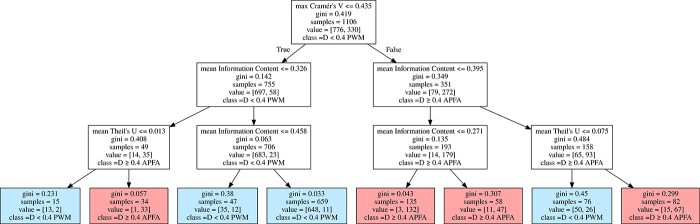
Decision tree to choose the best model. The leaves represent model preference based on Cohen’s D (D) measure calculated between APFA and PWM performance for each TFBS sample, where APFA is preferred when D ≥ 0.4 and PWM otherwise. The label “gini” refers to the gini impurity, “samples” is the total number of TFBS samples considered before a decision rule is made, “value” is the number of TFBS samples that reach that node. The final level of a Decision Tree is shown (pruned at level 3), where blue leaves represent the majority of TFBS samples classified as PWM-preferred and red leaves the APFA-preferred, showing overall good results.

Next, a Decision Tree classifier was learned to predict the best model (according to the two categories based on Cohen’s D) based on the same features. Figure 5 shows the decision tree, with depth limited to three levels, providing an average accuracy of 0.91 (standard deviation of ±0.03). This tree uses only the three features presenting the highest importance values: max_Cramér’s V (feature importance 0.696), mIC (feature importance 0.208) and mean_Theil’s U (feature importance 0.096). This result is coherent with the distribution of each feature in the two categories of model preference (see Figure S3).

**Chart 1 -:**
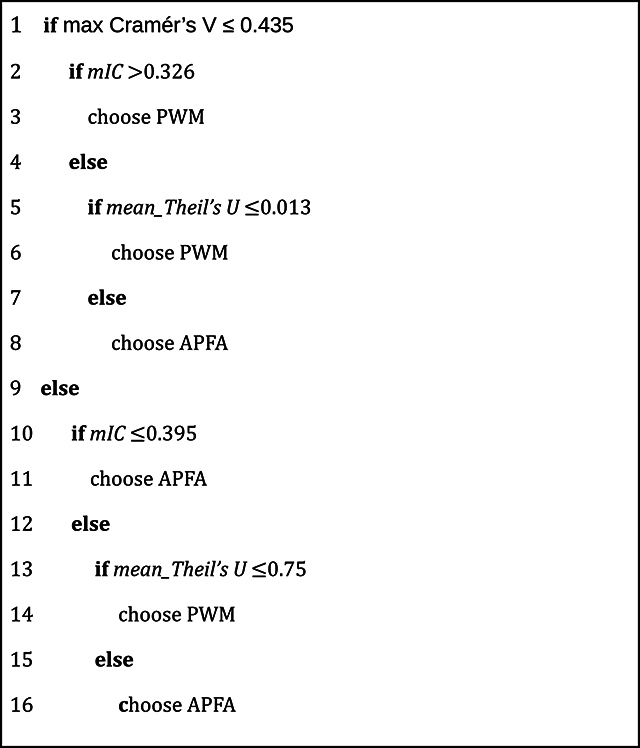
Decision Rules to choose between PWM and APFA. The algorithm shows how to choose between models PWM and APFA based on three extracted features, which were calculated using the TFBS sample.

Based on Figure 5, we propose the decision rule described at Chart 1 to choose the most appropriate model to a given TFBS sample based on only these three easily computable features - max Cramér’s V, mean Information Content and mean Theil’s U.

## Conclusion

This study showed that acyclic probabilistic finite automata (APFAs) are, in general, better suited models than position position weighted matrices (PWMs) when the TFBS sample has some amount of position dependency. Moreover, we propose a decision rule to choose with high accuracy which model to use, APFA or PWM, based on three relatively simple features calculated based only on the TFBS sample.

For approximately 70% of the samples tested, PWM was an appropriate choice given that APFA performed similarly and no additional evidence showed the importance of a more robust model. Nevertheless, in the remaining cases, APFA significantly outperformed PWM.

Finally, it is noteworthy that the method proposed here to compare models and infer decision rules to choose the most suitable one for a given training sample can be applied to the other applications outside of the scope of biology.

## Supplementary material

The following online material is available for this article:

Data S1 - Motif discovery on ENCODE datasets.

Data S2 - Motif Discovery on PBM datasets.

Data S3 - Nested k-fold cross-validation.

Table S1 - Inner loop of 7-fold Cross Validation.

Table S2 - Outer loop of 7-fold Cross Validation.

Figure S1 - Violin plots show distributions for each TFBS dataset combined with a motif algorithm.

Figure S2 - Direct comparison between PWM and APFA performance for all TFBS sample categories.

Figure S3 - Extracted features compared between model preference.
