# Supplementary material for: Position Weight Matrix or Acyclic Probabilistic Finite Automaton: Which model to use? A decision rule inferred for the prediction of transcription factor binding sites
Source: Genet Mol Biol. 2024 Jan 19;46(4):e20230048. doi: 10.1590/1678-4685-GMB-2023-0048 (PMC10945726; doi:10.1590/1678-4685-GMB-2023-0048)
Supplement: Data S1 - [file 1415-4757-GMB-46-4-e20230048-s1.pdf]

Genetics and Molecular Biology

https://doi.org/10.1590/1678-4685-GMB-2023-0048

Supplementary Material to "Position Weight Matrix or Acyclic Probabilistic Finite Automaton: Which model to use? A decision rule inferred for the prediction of transcription factor binding sites"

Data S1 - Motif discovery on ENCODE datasets

For ENCODE ChIP-seq datasets, narrowPeak files were converted to FASTA using BedTools (Quinlan and Hall, 2010) and masked genomes. From each narrowPeak file, sequences were extracted with 101 and 501 nucleotides (nt) centered at peak summit (i.e. the most intense signal value found in each peak).

RSAT and STREME. An adaptation of JASPAR protocol (supplementary file from Fornes et al., 2019) for recovering TFBS from ChIP-seq was used for each TF. ENCODE motifs were discovered by applying RSAT (Nguyen et al., 2018) and STREME (Bailey, 2021) in the 101 nt sequences. In order to pick the best motif, an enrichment analysis was applied in the 501 nt sequences using CENTRIMO (Bailey and Machanick, 2012) and all motifs discovered in the previous step. The motif with the lowest E-value (enrichment-value) was chosen and TFBSs were recovered using FIMO (Grant et al., 2011).

Top 15% of TFBS ranked by log-odd score and having p-value < 0.05 were collected. RSAT was executed using parameters:

"-markov auto -disco oligos,dyads,positions,local words -nmotifs 2 -minol 6 -maxol 7 -no merge lengths -ci 25 -noov -2str -origin center". STREME was executed using default parameters.

InMoDe order 1 and order 2. The algorithm InMoDe (Eggeling, 2018) was applied in centered 101 nt peaks with parameters "denovo mo=ω n=10", where ω is either 1 or 2 representing dependencies order. InMode motif length was set equal to the best motif found using the RSAT-STREME protocol.

TFFM Order 1. Sequences of 101 nt were first filtered by their top 20% highest signal values. For each 101 nt FASTA file, TFFM (Mathelier and Wasserman, 2013) was initialized using the best motif found in RSAT-STREME protocol. Binding sites were recovered by scanning all 501 nt sequences available (not filtering by signal) with the fitted model and recovering all TFBS with score above 0.8 provided that at least 100 sequences were collected, otherwise we used the top 100 TFBSs found ranked by their score.

References

Bailey TL (2021) STREME: accurate and versatile sequence motif discovery. Bioinformatics 37:2834–2840.

Bailey TL and Machanick P (2012). Inferring direct DNA binding from ChIP-seq. Nucleic Acids Research 40:e128.

Fornes O, Castro-Mondragon JA, Khan A, van der Lee R, Zhang X, Richmond PA, Modi BP, Correard S, Gheorghe M, Baranašić D, et al. (2019) JASPAR 2020: update of the open-access database of transcription factor binding profiles. Nucleic Acids Research, 48:D87–D92.

Grant CE, Bailey TL, Noble WS (2011). FIMO: scanning for occurrences of a given motif. Bioinformatics, 27:1017–1018.

Mathelier A, Wasserman WW (2013). The next generation of transcription factor binding site prediction. PLoS Computational Biology 9:e1003214.

Nguyen NTT, Contreras-Moreira B, Castro-Mondragon JA, Santana-Garcia W, Ossio R, Robles-Espinoza CD, Bahin M, Collombet S, Vincens P, Thieffry D et al. (2018). RSAT 2018: regulatory sequence analysis tools 20th anniversary. Nucleic Acids Research, 46:W209–W214.

Quinlan AR and Hall IM (2010). BEDTools: a flexible suite of utilities for comparing genomic features. Bioinformatics 26:841–842.
