# Supplementary material for: Position Weight Matrix or Acyclic Probabilistic Finite Automaton: Which model to use? A decision rule inferred for the prediction of transcription factor binding sites
Source: Genet Mol Biol. 2024 Jan 19;46(4):e20230048. doi: 10.1590/1678-4685-GMB-2023-0048 (PMC10945726; doi:10.1590/1678-4685-GMB-2023-0048)
Supplement: Data S2 - [file 1415-4757-GMB-46-4-e20230048-s2.pdf]

Genetics and Molecular Biology

https://doi.org/10.1590/1678-4685-GMB-2023-0048

Supplementary Material to "Position Weight Matrix or Acyclic Probabilistic Finite Automaton: Which model to use? A decision rule inferred for the prediction of transcription factor binding sites"

Data S2 - Motif Discovery on PBM datasets

Raw PBM files were ranked by their signal intensity and the top 1000 sequences from each replicate were further processed, totaling 2000 sequences.

RSAT and STREME. The motif with the lowest E-value (enrichment value) was selected for each TFBS sample (Bailey and Grant, 2021). Binding sites were recovered using FIMO (Grant et al., 2011). The top 15% TFBS ranked by log-odd score were considered, provided there were at least 100 sequences, otherwise the top 100 highest score TFBS were considered.

InMoDe order 1 and InMode order 2. InMoDe was applied using parameters "denovo n=10" and "mo=2" for order 2 and "mo=1" for order 1. Motif length was set according to the length of the best motif found by RSAT and STREME protocol for PBM. All binding sites returned by the algorithm were considered.

TFFM order 1. TFFM was applied with default parameters and initialized with best motif found by RSAT and STREME protocol for PBM. Binding sites were recovered scanning all 2000 sequences with the fitted model and recovering all TFBS with score above 0.8 provided that at least 100 sequences were collected, otherwise we used the top 100 TFBS ranked by score.

8-mer align E. We adapted the 8-mer align E algorithm (see Weirauch et al., 2013). First, all 8-mers E-scores according to their replicates "HK" and "ME". Then, 8-mers whose averages were above 0.45 were considered instances of motif and therefore were aligned with Clustal Omega (Sievers et al, 2011). We kept sequence positions that contained at least half nt count occupied. Gaps resulting from alignment were filled with a empirical distribution estimated from the

frequency of bases at the same considered position. Each unique 8-mer was replicated (E-score × 100) times and considered as TFBSs. E-score is a statistical measure that summarizes the intensity with which TF is bound to any 8-mer. For further details on that computation, see Berger and Bulyk (2009); Weirauch et al. (2013).

References

Berger MF and Bulyk ML (2009). Universal protein-binding microarrays for the comprehensive characterization of the DNA-binding specificities of transcription factors. Nature Protocols 4 :393–411.

Grant CE, Bailey TL, Noble WS (2011). FIMO: scanning for occurrences of a given motif. Bioinformatics, 27:1017–1018

Sievers F, Wilm A, Dineen D, Gibson TJ, Karplus K, Li W, Lopez R, McWilliam H, Remmert M, Söding J, Thompson JD and Higgins DG (2011). Fast, scalable generation of high-quality protein multiple sequence alignments using clustal omega. Molecular Systems Biology 7:539

Weirauch MT, Cote A, Norel R, Annala M, Zhao Y, Riley TR, Saez-Rodriguez J, Cokelaer T, Vedenko A, Talukder S, et al (2013) Evaluation of methods for modeling transcription factor sequence specificity. Nature Biotechnology 31:126–134.
