# Supplementary material for: Position Weight Matrix or Acyclic Probabilistic Finite Automaton: Which model to use? A decision rule inferred for the prediction of transcription factor binding sites
Source: Genet Mol Biol. 2024 Jan 19;46(4):e20230048. doi: 10.1590/1678-4685-GMB-2023-0048 (PMC10945726; doi:10.1590/1678-4685-GMB-2023-0048)
Supplement: Data S3 - [file 1415-4757-GMB-46-4-e20230048-s3.pdf]

Genetics and Molecular Biology

https://doi.org/10.1590/1678-4685-GMB-2023-0048

# Supplementary Material to "Position Weight Matrix or Acyclic Probabilistic Finite Automaton: Which model to use? A decision rule inferred for the prediction of transcription factor binding sites"

**Data S3** - Nested k-fold cross-validation

Tables S1 and S2 describe the first step ("inner loop") and second step ("outer loop") of the nested K-fold cross-validation described at section Material and Methods – Model performance evaluation, topic Nested k-fold CV.
