# Supplementary material for: Position Weight Matrix or Acyclic Probabilistic Finite Automaton: Which model to use? A decision rule inferred for the prediction of transcription factor binding sites
Source: Genet Mol Biol. 2024 Jan 19;46(4):e20230048. doi: 10.1590/1678-4685-GMB-2023-0048 (PMC10945726; doi:10.1590/1678-4685-GMB-2023-0048)
Supplement: Table S1 - [file 1415-4757-GMB-46-4-e20230048-s4.pdf]

Genetics and Molecular Biology

https://doi.org/10.1590/1678-4685-GMB-2023-0048

Supplementary Material to “Position Weight Matrix or Acyclic Probabilistic Finite Automaton: Which model to use? A decision rule inferred for the prediction of transcription factor binding sites”

Table S1 - Hyperparameter calibration and threshold selection for K-fold

CV step 1. The Table S1 exemplifies the algorithm to train and calibrate both APFA and PWM models.

Input:

k: number of folds

S^+: positive sample

S^-: negative sample

model: indicates if model is APFA or PWM

Output:

c*: optimal Learn-APFA hyperparameters

t*: optimal APFA or PWM threshold

procedure Calibration step(k, $S+$, $S-$, model)
    divide $S+$ in k disjoint sets ($fold^+$) of equal size (or the closest possible)
    divide $S-$ in k disjoint sets ($fold^-$) of equal size (or the closest possible)
    for each i, with $i = 0, 1, 2, ..., k-1$
        define *test* fold as $fold^+$ i and $fold^-$ i
        define *calibration* fold as $fold^+$ $(i + 1) \bmod k$ and $fold^-$ $(i + 1) \bmod k$
        define training folds as $\bigcup_{j \in \{0,1,2,...,k-1 | j \notin \{i,i+1\}\}} fold^+ j$
        if the *model* is APFA, then
            for each combination *c* of hyperparameters:
                train models using *c* and *training* folds
                compute AP score over *calibration* fold
                find optimal classification threshold t_i* which maximizes F1-score
        if the *model* is PWM, then
            train models using *training* folds

```
        compute AP score over calibration folds
        find optimal classification threshold t_i* which maximizes F1-score
  compute the average AP
if the model is APFA, then
    c* ← hyperparameter combination that maximizes the average AP
t* ← average of all t_i* from each fold i.
```
