# Supplementary material for: Position Weight Matrix or Acyclic Probabilistic Finite Automaton: Which model to use? A decision rule inferred for the prediction of transcription factor binding sites
Source: Genet Mol Biol. 2024 Jan 19;46(4):e20230048. doi: 10.1590/1678-4685-GMB-2023-0048 (PMC10945726; doi:10.1590/1678-4685-GMB-2023-0048)
Supplement: Table S2 - [file 1415-4757-GMB-46-4-e20230048-s5.pdf]

Genetics and Molecular Biology

https://doi.org/10.1590/1678-4685-GMB-2023-0048

Supplementary Material to "Position Weight Matrix or Acyclic Probabilistic Finite Automaton: Which model to use? A decision rule inferred for the prediction of transcription factor binding sites"

Table S2 - Model performance evaluation step for the nested K-fold CV. The Table S2 exemplifies the algorithm to test both PWM and APFA models in order to choose the best fitting model. Notice that the test fold is utilized exclusively for this algorithm.

Input:

k: number of folds (the same used in Table S1)

S^+: positive sample (the same used in Table S1)

S^-: negative sample (the same used in Table S1)

model: indicates if model is APFA or PWM

c*: hyperparameter combination calibrated in Table S1 (for APFA)

t*: optimal threshold calibrated in Table S1

Output:

model AP score and additional performance measures based on t*

procedure Performance evaluation step (*k*, S^+, S^-, *model*, c*, t*)

  use the same fold division fold+ e fold− of Table S1 for S^+ and S^-

  for each i, with i = 0, 1, ..., *k* − 1

    define *test* fold as fold+ i and fold− i

    define *training* folds as $\bigcup_{j \neq i}$ fold + j

    if the is model APFA, then

      train model using *training* folds and c*

    if the is model PWM, then

      train model using *training* folds

    use the trained model to compute the scores of *test* fold

    compute PR curve and respective AP

    use t* to compute additional measures such as accuracy, F1-score, etc.

  compute the average value for AP and for the additional measures
